# Sports or no sports? How to manage the young arrhythmia patient

**DOI:** 10.1007/s00399-025-01089-3

**Published:** 2025-07-28

**Authors:** Ilger Ertugrul, Nico A. Blom

**Affiliations:** 1 Pediatric Cardiology, Department of Pediatrics, Ihsan Dogramaci Children’s Hospital, Hacettepe University, Ankara, Turkey; 2 Department of Pediatric Cardiology, Amsterdam University Medical Center, Amsterdam, The Netherlands; 3 Department of Pediatric Cardiology, Leiden University Medical Center, Leiden, The Netherlands

**Keywords:** Inherited cardiac diseases, Pediatrics, Sports participation, Sudden cardiac death, Shared decision-making, Erbliche Herzerkrankungen, Pädiatrie, Teilnahme am Sport, Plötzlicher Herztod, Gemeinsame Entscheidungsfindung

## Abstract

**Background:**

Inherited arrhythmia syndromes and cardiomyopathies are among the most concerning causes of sudden cardiac death in young individuals, particularly in the context of physical activity. Historically, sports participation in these patients has been broadly restricted due to safety concerns. However, emerging data and updated guidelines suggest that a more individualized approach may be both appropriate and safe.

**Objectives:**

The aim of this study was to review current evidence and evolving recommendations regarding sports participation in young individuals with inherited cardiac diseases.

**Materials and methods:**

This review synthesizes recent studies, expert consensus statements, and current international guidelines (ESC, AHA/ACC).

**Results:**

Recent data indicate that, in selected patients with inherited arrhythmia syndromes and cardiomyopathies who have undergone thorough evaluation and counseling, participation in sports—under appropriate precautions—may be safe and well tolerated. Emerging studies report low incidence of adverse events in appropriately managed athletes. Guidelines have shifted away from blanket restrictions and towards shared decision-making, especially in asymptomatic individuals or those with controlled disease. Key factors include genotype-phenotype correlation, history of arrhythmic events, treatment adherence, and patient/family understanding of risks.

**Conclusion:**

In contrast to traditional dogma, a growing body of evidence supports less restrictive, patient-centered management for young individuals with inherited cardiac conditions. With proper evaluation, risk stratification, and informed decision-making, sports participation and leisure time activities may be possible—and even beneficial—for many of these patients.

## Abstract (de)

**Hintergrund:**

Erbliche Arrhythmiesyndrome und Kardiomyopathien gehören zu den besorgniserregendsten Ursachen des plötzlichen Herztods bei jungen Menschen, insbesondere im Zusammenhang mit körperlicher Aktivität. Aus Sorge um die Sicherheit wurden sportliche Aktivitäten für betroffene Patienten in der Vergangenheit oft weitgehend eingeschränkt. Neue Daten und aktualisierte Leitlinien deuten jedoch darauf hin, dass ein individuelleres Vorgehen sowohl angemessen als auch sicher sein kann.

**Ziel der Arbeit:**

Ziel dieses Beitrags ist es, die aktuelle Evidenz und die sich wandelnden Empfehlungen zur Sportbeteiligung junger Menschen mit erblichen Herzerkrankungen darzustellen.

**Material und Methoden:**

Diese Übersicht basiert auf der Analyse aktueller Studien, Expertenempfehlungen und internationaler Leitlinien (European Society of Cardiology [ESC], American Heart Association [AHA]/American College of Cardiology [ACC]) zu erblichen Arrhythmiesyndromen und Kardiomyopathien.

**Ergebnisse:**

Neue Daten zeigen, dass die Teilnahme an sportlichen Aktivitäten – unter angemessenen Vorsichtsmaßnahmen – bei ausgewählten Personen mit erblichen Arrhythmiesyndromen und Kardiomyopathien nach umfassender Evaluation und Aufklärung sicher und gut verträglich sein kann. Studien berichten über eine niedrige Inzidenz unerwünschter Ereignisse bei adäquat betreuten Athleten. Die aktuellen Leitlinien bewegen sich weg von generellen Sportverboten und hin zu einem Modell der gemeinsamen Entscheidungsfindung, insbesondere bei asymptomatischen Personen oder solchen mit gut kontrollierter Erkrankung. Entscheidende Faktoren sind unter anderem die Genotyp-Phänotyp-Korrelation, die Vorgeschichte arrhythmischer Ereignisse, die Therapietreue sowie das Risikoverständnis der Patienten und ihrer Familien.

**Schlussfolgerung:**

Im Gegensatz zur bisherigen Lehrmeinung unterstützt eine wachsende Evidenz ein weniger restriktives, patientenzentriertes Management junger Menschen mit erblichen Herzerkrankungen. Bei sorgfältiger Evaluation, Risikostratifizierung und informierter Entscheidungsfindung kann Sport einschließlich Freizeitaktivitäten für viele dieser Personen möglich und sogar vorteilhaft sein.

## Introduction

Young people have an intrinsic drive to engage in sports and leisure activities, driven by a combination of biological, psychological, and social factors. Hormonal changes during adolescence—such as increased testosterone and dopamine levels—enhance energy and motivation. Physical activity also stimulates the release of endorphins, promoting pleasure and reducing stress. Physical activity also provides a sense of achievement, confidence, and physical well-being, while improving health. The desire for acceptance and recognition within their social circles plays a crucial role [6].

Sudden cardiac death (SCD), especially in inherited arrhythmia syndromes and cardiomyopathies, is often associated with adrenergic stimulation. Until now, most treatment approaches—except in special circumstances—have focused on blocking the adrenergic system. Consequently, lifestyle modifications, particularly limiting physical activities, have been widely recommended to improve survival. As a result, patients are often restricted from both daily physical activities and participation in competitive sports. Physical activity has been associated with SCD, which has a devastating impact on both families and society. In response, preparticipation athlete screenings have been implemented to identify vulnerable individuals, leading to significant reductions in sudden deaths. Considering that it is nearly impossible to completely prevent young individuals from engaging in sports and physical activities, focus should shift from restriction to facilitating safe participation [10]. The distinction between leisure activities and competitive sports is often unclear, as both can involve similar levels of intensity and exertion. It is also impossible to quantify limits of activity on the field or street. Factors such as activity duration, environmental temperature, nutrition, and hydration can significantly influence the intensity and risk of the same activity.

The 36th Bethesda Conference guidelines traditionally imposed strict restrictions on sport participation across all patient subgroups [25]. Considering inherited arrhythmias, which are classified as rare diseases with an incidence of 1 in 2000 [35], and inherited cardiomyopathies, such as hypertrophic cardiomyopathy (1 in 500), and arrhythmogenic cardiomyopathy (1 in 5000) [24], these restrictions affect over 40,000 young individuals. This number continues to rise due to advances in genetic screenings that identify more asymptomatic gene carriers. In 2006, the American Heart Association/American College of Cardiology/European Society of Cardiology (AHA/ACC/ESC) guideline on ventricular arrhythmia management and SCD prevention recommended against competitive sports participation for individuals with inherited arrhythmias and cardiomyopathies. These recommendations were largely based on expert consensus with low levels of evidence. Nearly a decade later, AHA guidelines presented new strategies for managing patients with cardiac ion channelopathies in the context of sports participation, emphasizing individualized evaluation by heart rhythm specialists or genetic cardiologists, with management tailored to their clinical presentation. Most recently, in 2024, The Heart Rhythm Society (HRS) published a consensus statement on the evaluation, treatment, and return-to-play eligibility for athletes. This highlighted the importance of shared decision-making (SDM) in managing individuals with genetic arrhythmia syndromes and cardiomyopathies [11, 21, 25].

For these patients, the decision to participate in sports should be individualized. High-risk groups require customized management. Low-risk patients should not be treated with the same caution as high-risk individuals—although they are not risk-free. Determining sports eligibility should involve collaborations between healthcare providers and patients using an SDM framework [21]. SDM integrates clinical evidence with patient values, preferences, and goals, ensuring that medical decisions align with the individual’s unique circumstances. For minors, parents play a vital role in this process—particularly in decisions surrounding sports participation for at-risk individuals. Their involvement ensures that the athlete’s preferences and the psychological impact of potential restrictions are fully considered. It is essential that families are thoroughly informed about the risks of SCD, the psychological consequences of limiting participation, and the potential benefits of physical activity.

For SDM to be effective, several key components are necessary:

1. Evidence-based information: Patients and families should be provided with accurate, up-to-date medical information, including the risks, benefits, and alternatives to participation (typically derived from clinical guidelines and societal statements).
2. Patient engagement: Patients should be encouraged to express their values, concerns, and preferences related to treatment and activity options.
3. Deliberation and discussion: A structured dialogue between clinicians, patients, and families where all options are carefully weighed.
4. Joint decision-making: A collaborative agreement that balances clinical recommendations with patient and family priorities.

**Fig. 1:**
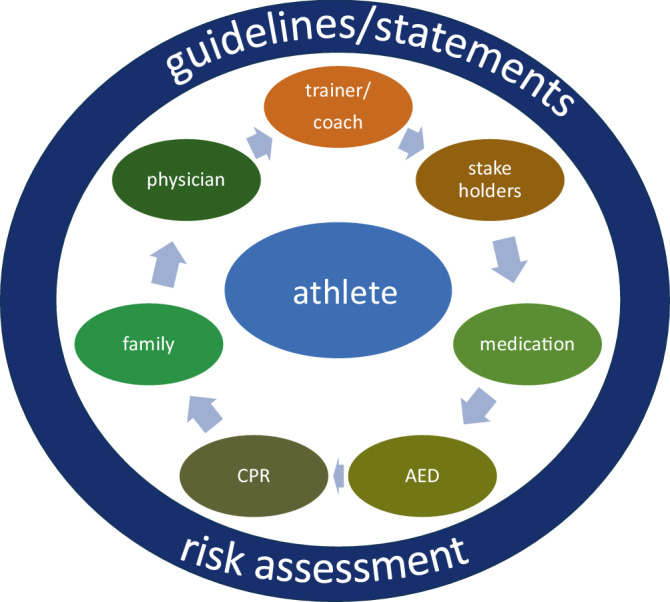
Model for shared decision-making for young athletes with inherited cardiac diseases. *CPR* cardiopulmonary resuscitation, *AED* automatic external defibrillator

In addition, young individual and parents should be updated on any new information that may influence decision-making. This process should also extend beyond the family and clinical team. Coaches, facility medical staff, institutional stakeholders, and teammates should also be included to ensure a unified and supportive environment. Another critical safety measure is the presence of personnel trained in basic life support (BLS), and immediate access to an automated external defibrillator (AED). Early defibrillation is the most significant determinant of survival following sudden cardiac arrest (SCA), with survival rates declining by 7–10% for every minute of delay [7]. To minimalize delays, a comprehensive, rehearsed emergency action plan should be in place, ensuring efficient and coordinated response [8]. Both the individual and their surrounding environment must be prepared to prevent or respond effectively to adverse events. Adherence to the treatment plan is another crucial factor in determining eligibility for sports participation and is strongly influenced by the SDM process. Among young individuals with chronic illnesses, adherence rates vary widely (between 10 and 89%). However, SDM has been shown to improve compliance, in particularly in athletes who are motivated to continue participating in sports. One key contributor to improved adherence is the athlete’s clear understanding of their diagnosis, the associated risks, and the rationale for their treatment plan. This process is closely linked to SDM and the motivation to sports participation ([10, 39]; Fig. 1).

This review explores the sports participation and leisure time activities of young individuals with inherited arrhythmia syndromes and cardiomyopathies. It emphasizes strategies to ensure safe engagement in physical activity and outlines the precautions necessary to minimize risk.

## Inherited arrhythmia syndromes

A significant proportion of SCA and SCD are associated with inherited arrhythmia syndromes, including congenital long QT syndrome (LQTS), Brugada syndrome (BrS), catecholaminergic polymorphic ventricular tachycardia (CPVT), and short QT syndrome (SQTS). Making decisions regarding exercise restriction for individuals with these diseases is challenging for both patients and physicians, and even more so for competitive and non-competitive athletes.

## Long QT syndrome

LQTS is the most common cardiac channelopathy, with an estimated incidence of 1 in 2000 individuals. This repolarization abnormality predisposes patients to ventricular tachycardia, particularly torsades de pointes, which may lead to SCD. Most LQTS subtypes respond well to beta-blocker therapy, with the notable exception of LQTS type 3. Preventive strategies are universally recommended and include avoidance of QT-prolonging medications and correction of electrolyte imbalances. In recent years, left cardiac sympathetic denervation (LCSD) has gained attention as a viable treatment option by effectively blocking the sympathetic innervation of the heart. While the prognosis for LQTS type 3 is generally poorer than for type 1, the risk of cardiac events during physical activity is very low. LQTS1, on the other hand, carries the highest risk for exercise-induced cardiac events among the subtypes [34].

Historically, young patients with LQTS were discouraged from sports participation due to the presumed high risk, despite the low level of evidence. However, emerging data suggest a more nuanced view. A study of 352 patients (mean age 11 ± 7 years) demonstrated that medication adherence is the most critical factor in preventing arrhythmias. Notably, no sport-related cardiac events were reported over 650 patient-years of follow-up [17]. Additionally, a return-to-play program involving 494 patients (mean age at diagnosis: 14.8 ± 10.5 years; mean follow-up: 4.2 ± 4.8 years) reported an event rate of only 1.16 non-lethal events per 100 athlete-years. Importantly, most breakthrough cardiac events were not associated with sports participation. Among 354 patients in that cohort, no correlation was found between sport type and event occurrence. The most commonly played sports were basketball (27%), soccer (17%), volleyball (8%), and swimming (8%), with 34% of athletes participating in multiple sports [39]. Managing asymptomatic individuals with mildly prolonged QTc intervals—often diagnosed through family or preparticipation screening—is another key consideration. While no multicenter studies have addressed this population specifically, a Mayo Clinic study reported a very low event rate of 0.18 events per 100 patient-years in return-to-play athletes without an implantable cardioverter-defibrillator (ICD), indicating a lower-risk subgroup [38].

**Fig. 2:**
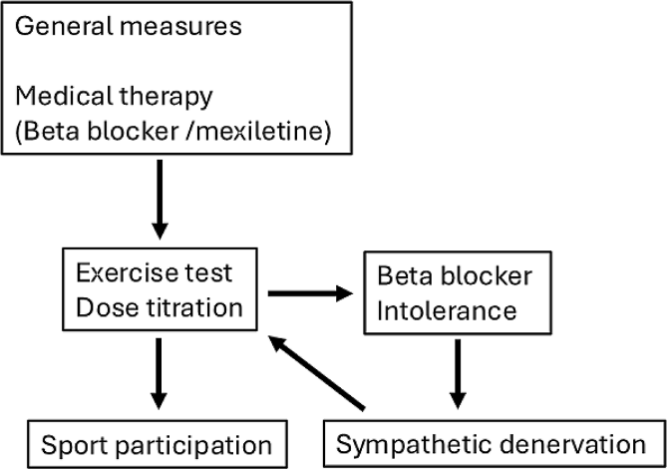
Algorithm for sports participation in long QT syndrome

These findings have informed recent changes in clinical practice, as reflected in both the ESC position paper on physical activity and sports participation for LQTS patients, and the 2024 HRS expert consensus statement on arrhythmias in athletes [15, 21]. These guidelines emphasize SDM, medication dose adjustments, and the roles of pharmacotherapy and LCSD. They also recommend exercise testing as part of preparticipation evaluations, although a negative test should not be viewed as reassuring, as emotional stress may also trigger events [16]. Preventive strategies remain crucial, including the availability of an AED at sporting events—particularly for higher-risk patients. Special caution is warranted for swimming in LQTS1, a known trigger, especially in untreated individuals. For those on medical therapy, the risk is reduced, though precautions—such as avoiding swimming alone or in open water and ensuring AED access—remain essential. Finally, clinicians must remain vigilant during decision-making. Over 60% of cardiac arrests in LQTS occur as the first manifestation of the disease in previously asymptomatic individuals. Furthermore, remaining event-free until the age of 18–20 does not guarantee lifelong protection ([33]; Fig. 2).

## Catecholaminergic polymorphic ventricular tachycardia

Catecholaminergic polymorphic ventricular tachycardia (CPVT) is a rare inherited arrhythmia that occurs in structurally normal hearts, with an incidence of 1 in 10,000. It is characterized by the appearance of bidirectional ventricular ectopy at heart rates of 110–120 bpm, while the resting electrocardiogram (ECG) remains normal [2]. Diagnosis is typically confirmed through an exercise test that demonstrates bidirectional extrasystoles increasing with heart rate, although this finding may not always be present [31]. CPVT is triggered by increased sympathetic tone, such as during exercise or emotional stress, and results from abnormalities in intracellular calcium handling [5]. First-line treatment for CPVT includes beta-blockers, either alone or in combination with flecainide. LCSD may be considered as an additional treatment for CPVT. For cardiac arrest survivors or patients who remain symptomatic despite maximal therapy, ICD implantation is recommended. A point of debate remains whether cardiac arrest survivors should first receive optimal medical therapy with beta-blockers and flecainide before considering ICD implantation Proper ICD programming is important to prevent electrical storms caused by catecholamine surges following multiple shocks [32].

**Fig. 3:**
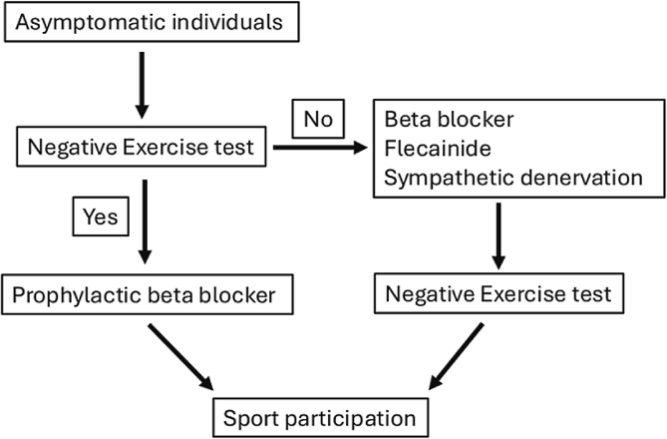
Algorithm for sports participation in catecholaminergic polymorphic ventricular tachycardia

Due to its nature, CPVT carries a high risk of exercise-induced cardiac events, and exercise restriction is generally advised. In a Mayo Clinic series on athletes with genetic heart diseases who elected to return to play, a small subset had CPVT. Among these, the high-risk group with an ICD (*n* = 17) and the lower-risk group without an ICD (*n* = 44) showed non-lethal event rates of 4.9 and 0.19 per 100 athlete-years, respectively. In general, sports participation may be considered for asymptomatic individuals through a SDM process and with appropriate therapy (Fig. 3. In contrast, symptomatic patients should be restricted from sports [16]. The effectiveness of medical therapy should be evaluated using exercise testing to confirm that ventricular arrhythmias are suppressed during activity. The primary therapeutic goal is complete elimination of ectopy. While bigeminal ventricular extrasystoles may be tolerated, couplets indicate a need for intensification of therapy. If available, nadolol is the preferred beta-blocker; otherwise, propranolol is recommended. Beta-blocker therapy should be increased in small dose adjustments for better tolerability. Flecainide should be considered as part of combination therapy. If arrhythmias are not controlled on exercise testing despite medical therapy, or if beta-blockers are not tolerated, LCSD should be considered. If adverse events occur despite all treatment efforts, sports participation should be prohibited [21, 27].

## Brugada syndrome

Brugada syndrome (BrS) is a rare inherited cardiac channelopathy characterized by a distinct ECG (type I Brugada pattern) and an increased risk of ventricular arrhythmias and SCD, particularly in young males. In children, BrS is very rare, and symptoms most commonly occur during fever, predominantly in those with an *SCN5A* mutation [16, 30]. Symptoms are rarely linked to elevated heart rates, although in certain genetic variants, exercise testing may unmask the characteristic ECG pattern and potentially induce ectopy. Although there are no conclusive data indicating a high risk during exercise in individuals with BrS, increases in body temperature, particularly in warm climates, should be taken into account. Additional precautions include avoiding alcohol, heavy meals, marijuana, and medications that act on sodium channels. There are no specific restrictions on sports participation for patients with BrS. Even individuals who have undergone ICD implantation may be considered for sports participation under SDM protocols, provided at least 3 months have passed since implantation [16, 19].

## Short QT syndrome

Short QT syndrome has only been defined in recent years, and therefore, data on sports participation remain limited. Although only approximately 15% of cardiac events are reported to be triggered by adrenergic stimuli—with most occurring at rest or during sleep—the European Heart Rhythm Association (EHRA) position paper recommends restricting sports participation, with the possible exception of leisure activities. However, the more recent HRS Consensus Statement on arrhythmias in athletes suggests that preventive measures, such as avoiding electrolyte disturbances and administering quinidine in symptomatic patients or in those with a QTc interval shorter than 320 ms, may allow for unrestricted physical activity [3, 21].

## Inherited cardiomyopathies

Inherited cardiomyopathies, although often asymptomatic and not associated with overt deterioration of cardiac function, can feature a disorganized myocardial structure, placing affected individuals at high risk for arrhythmias and SCD, particularly during sports participation. Among these, arrhythmogenic cardiomyopathy (ACM) and hypertrophic cardiomyopathy (HCM) are the leading causes of exercise-related SCD in Italy and the USA, respectively. The phenotypic expression of these cardiomyopathies can vary widely, highlighting the importance of individualized risk stratification. In line with evolving perspectives on inherited arrhythmia syndromes, recent evidence—such as long-term follow-up data of HCM patients who continued sports participation without a high incidence of adverse events [28]—has prompted a shift in clinical approach. Whereas earlier guidelines recommended exclusion from competitive sports [25], more recent guidance supports expert-led evaluation and an SDM model for determining sports eligibility in this population [16, 21].

## Hypertrophic cardiomyopathy

Hypertrophic Cardiomyopathy (HCM) is the most common form of cardiomyopathy associated with SCD, accounting for 36% of SCD in athletes [22]. Due to this significant risk, the Bethesda Conference previously recommended strict restrictions on athletic participation for individuals with HCM [25]. HCM is characterized by hypertrophied ventricular myocardium, but pathologic specimens revealed more than just increased myocardial mass. In fact, it involves disruption of myocardial architecture and replacement fibrosis, leading to myocardial disarray. This disorganization predisposes patients to ventricular arrhythmias more so than systolic dysfunction itself. While earlier studies showed that 16% of SCD in athletes with HCM occurred during exercise [23], more recent studies suggest a lower incidence of SCD. For example, one study found that only 1.7% of participants experienced ventricular arrhythmias during exercise [12]. A recent large multicenter cohort study of 1660 patients, including pediatric patients, showed that individuals with HCM or those who are genotype-positive/phenotype-negative, when managed in experienced centers, did not have higher rates of SCD or life-threatening arrhythmias with vigorous exercise compared to those with moderate or sedentary activity levels [20]. These finding suggest that guidance on sports and physical activity for HCM patients may become less restrictive moving forwards. Additionally, childhood exercise has shown benefits in preserving diastolic function—including relaxation, filling, and cavity size [4]—and avoiding the detrimental effects of a sedentary life style [18]. Consequently, over-restricting physical activity in young HCM patients may actually negatively impact long-term cardiovascular health.

A critical step in the SDM process is the risk stratification of patients. Risk factors for HCM-related SCD have been well defined, and validated risk calculators exist for both adults and children (https://hcmriskkids.org). An algorithm was proposed for sport participation in HCM, categorizing patients into risk groups and suggesting activity levels accordingly [36]. The LIVE-HCM study uses clinical parameters such as symptom burden, history, left ventricular outflow tract (LVOT) gradients, blood pressure response, exercise-induced arrhythmias, and functional capacity to stratify individuals as low-moderate and high risk and give suggestions according to that classification ([36]; Table 1). Patients with symptomatic obstructive HCM should undergo therapeutic interventions to alleviate left ventricular outflow tract obstruction before return to play is considered [21]. According to the 2024 HRS consensus statement, individuals who are genotype-positive but phenotype-negative are allowed to resume athletic activity [21]. However, because phenotypic expression of HCM is age-dependent, serial follow-up is essential, particularly in younger athletes, to monitor for disease progression over time [13].

**Table 1:** Exercise participation by hypertrophic cardiomyopathy (HCM) risk category

Risk category	Clinical features	Exercise participation recommendation
Low risk	Asymptomatic	Participation in high-intensity exercise is generally allowed
	LVOT gradient < 30 mm Hg	
	Normal blood pressure response	
	No arrhythmias	
Moderate risk	Symptoms not clearly related to exercise	Moderate-intensity exercise may be appropriate with individual assessment
	LVOT gradient 30–49 mm Hg	
	Attenuated BP response	
	Exercise-induced PVCs	
High Risk	History of cardiac arrest or syncope	Low-intensity exercise is recommended; therapeutic intervention required before return to play
	LVOT gradient a50 mm Hg	
	Systolic BP drop during exercise	
	Ventricular tachycardia	

*LVOT* Left ventricular outflow tract, *BP* Blood pressure, *PVCs* Premature ventricular contractions

## Arrhythmogenic cardiomyopathy

Arrhythmogenic cardiomyopathy (ACM) or if limited to the right ventricle defined as arrhythmogenic right ventricular cardiomyopathy, is a form of cardiomyopathy characterized by fibro-fatty replacement of ventricular myocardium. This disruption of myocardial architecture by adipose and fibrous tissue leads to ventricular arrhythmias, myocardial dysfunction, and SCD. Although the disease was initially described as involving primarily the right ventricle, it is now well established that both ventricles can be affected [14]. ACM is generally inherited in an autosomal dominant fashion, with most causative mutations affecting desmosomal genes. The disease typically manifests after the second decade of life, but it tends to present more frequently as SCA or SCD in pediatric patients compared to adults [37]. As a progressive disease, ACM leads to increasingly pronounced structural abnormalities over time. Notably, the silent phase—prior to overt heart failure—still carries a high risk of arrhythmias.

ACM is more commonly observed and manifests earlier in individuals who participate in sports. While exercise—particularly vigorous activity—is known to increase the risk of ventricular arrhythmias and SCD, it also significantly accelerates disease progression [14]. Conversely, low-intensity exercise, even over extended periods, does not appear to affect the natural course of the disease [36]. Genetic mutations play a critical role in risk stratification and SDM. Disease progression and arrhythmia risk are particularly high in individuals with *PKP2* mutations. For patients with non-*PKP2* mutations, such as Phospholamban (PLN) variants, data are limited; however, sports participation may be considered in those without overt phenotypic expression. For patients with a clear ACM phenotype, return-to-play decisions should be guided by both genotype and the intensity of physical activity. Those with high-risk phenotypes may benefit from restrictions, while low-risk genotypes may be cautiously considered for participation based on recent data. Special attention should be paid to younger patients, given the potential for long-term progression and arrhythmic risk [16, 21].

## Dilated Cardiomyopathy: left ventricular non-compaction

Dilated Cardiomyopathy (DCM) is a condition characterized by ventricular dilatation and functional impairment. Pathological examinations reveal disruption of the myocardial muscle structure and an increase in fibrosis within the connective tissue. Genetic mutations can be detected in approximately one-third of patients. In individuals engaged in intense sports, similar features—such as increased ventricular size and reduced functional measurements—may also be present, making the differentiation between physiological adaptation and DCM particularly challenging [1]. However, regional myocardial involvement, right ventricular dysfunction, and ECG abnormalities are more suggestive of DCM. In this context, cardiac magnetic resonance imaging (MRI) with late gadolinium enhancement (LGE) is a valuable tool for both differential diagnosis and risk assessment for ventricular arrhythmias (VA). Left ventricular non-compaction (LVNC) is a rare cardiomyopathy marked by an abnormally thick, trabeculated myocardium and a thin compact layer, primarily affecting the left ventricle. Although classified as a genetic cardiomyopathy, LVNC may also involve the right ventricle or both ventricles. It can be associated with structural heart anomalies and chromosomal abnormalities. However, its diagnosis is controversial due to the presence of similar myocardial patterns in up to 20% of healthy individuals, as well as in physiological conditions such as pregnancy and athletic remodeling. While frequently asymptomatic, LVNC can pose significant risks, including thromboembolic events, ventricular dysfunction, ventricular arrhythmias, and SCD [26, 40].

DCM is a rare but recognized cause of SCD in young competitive athletes. Epidemiological studies report its contribution to cardiovascular-related deaths as 4% in Italy, 2.5%–8% in the USA, and 1% in the UK. Importantly, isolated LVNC has not been reported as a direct cause of SCD in any of these athletic populations [41]. When left ventricular dilatation is the only finding, without additional indicators of DCM, the athlete may be allowed to participate in competitive sports. Similarly, asymptomatic athletes with no history of unexplained syncope, no frequent or complex ventricular arrhythmias on ambulatory ECG or exercise testing, and only mildly reduced ventricular function may be selectively cleared for participation in all types of sports. Notably, no major cardiac events have been reported in patients without ventricular dysfunction, regardless of the degree of hypertrabeculation [16]. Conversely, athletes diagnosed with DCM who are symptomatic, have a left ventricular ejection fraction (LVEF) below 40%, show extensive LGE on cardiac MRI, experience frequent or complex ventricular arrhythmias, or have a history of unexplained syncope should avoid vigorous exercise and competitive sports. In such cases, even leisure-time physical activity should be approached with caution and under close medical supervision [29].

## Conclusion

Traditionally, clinical decision-making around sports participation and leisure time activity has focused primarily on the risks associated with continued engagement in sports, particularly the potential for sudden cardiac death or other adverse cardiovascular events. As a result, eligibility guidelines have historically prioritized safety, frequently leading to very conservative restrictions, especially for young athletes with inherited or structural heart conditions. However, this risk-averse approach often fails to consider the equally important consequences of *not* participating. When restrictions are imposed, the potential physical, cognitive, emotional, and social consequences are frequently overlooked. Physical inactivity accounts for nearly 9% of premature mortality globally and is a major contributor to chronic disease. In children and adolescents, regular exercise is strongly associated with improved cardiovascular health, enhanced brain development, better cognitive function, and superior academic performance. Furthermore, restriction from sports participation can significantly impact quality of life and mental health, with studies indicating a 25% lower risk of depression in physically active individuals. The psychological toll of exclusion—including loss of identity, reduced resilience, and increased anxiety or depression—can be profound. Notably, depression and suicide together account for approximately 11% of youth mortality worldwide, posing a greater threat to adolescents than many cardiac conditions [9, 10]. Beyond mental health, restricted athletes, especially teenagers may turn to unsupervised or unregulated physical activity, often in settings with greater risk and no medical oversight. This unintended consequence can paradoxically increase the likelihood of harm. Ultimately, there is a delicate balance between the health benefits of exercise and the cardiac risks it may pose in individuals with inherited or acquired cardiovascular diseases. In some conditions, exercise may act as a trigger for life-threatening events; in others, it may contribute to disease progression. Therefore, risk assessment must go beyond a binary approach of participation versus restriction. Instead, it should adopt a nuanced, personalized strategy—ideally within a shared decision-making framework involving cardiologists, sports physicians, psychologists, the patient, and their family. This multidisciplinary model allows for a comprehensive evaluation of medical, psychological, and social factors, aiming to safeguard not only the athlete’s physical health but also their overall well-being.

## Declarations

For this article no studies with human participants or animals were performed by any of the authors. All studies mentioned were in accordance with the ethical standards indicated in each case.
